# Supplementary material for: A Cross-Sectional Study of the Relationship Between Dietary Micronutrient Intake, Cognition and Academic Performance Among School-Aged Children in Taabo, Côte d’Ivoire
Source: Nutrients. 2025 Nov 18;17(22):3602. doi: 10.3390/nu17223602 (PMC12655121; doi:10.3390/nu17223602)
Supplement: Supplementary file 1 [file nutrients-17-03602-s001.zip › supplementary Figures S1-S3.pdf]

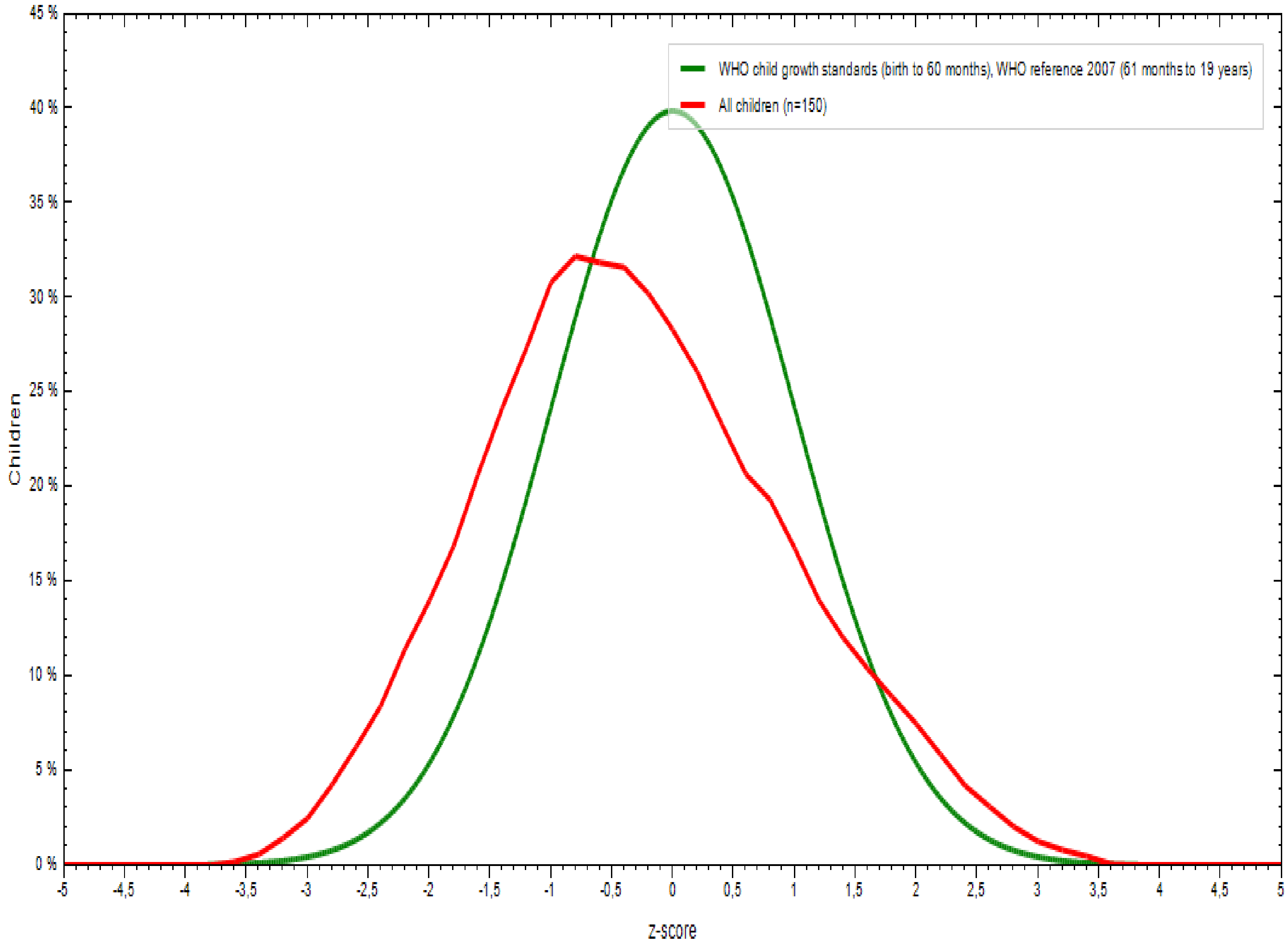

Figure S1. Comparison of participants BMI-for-age values with WHO growth standards.

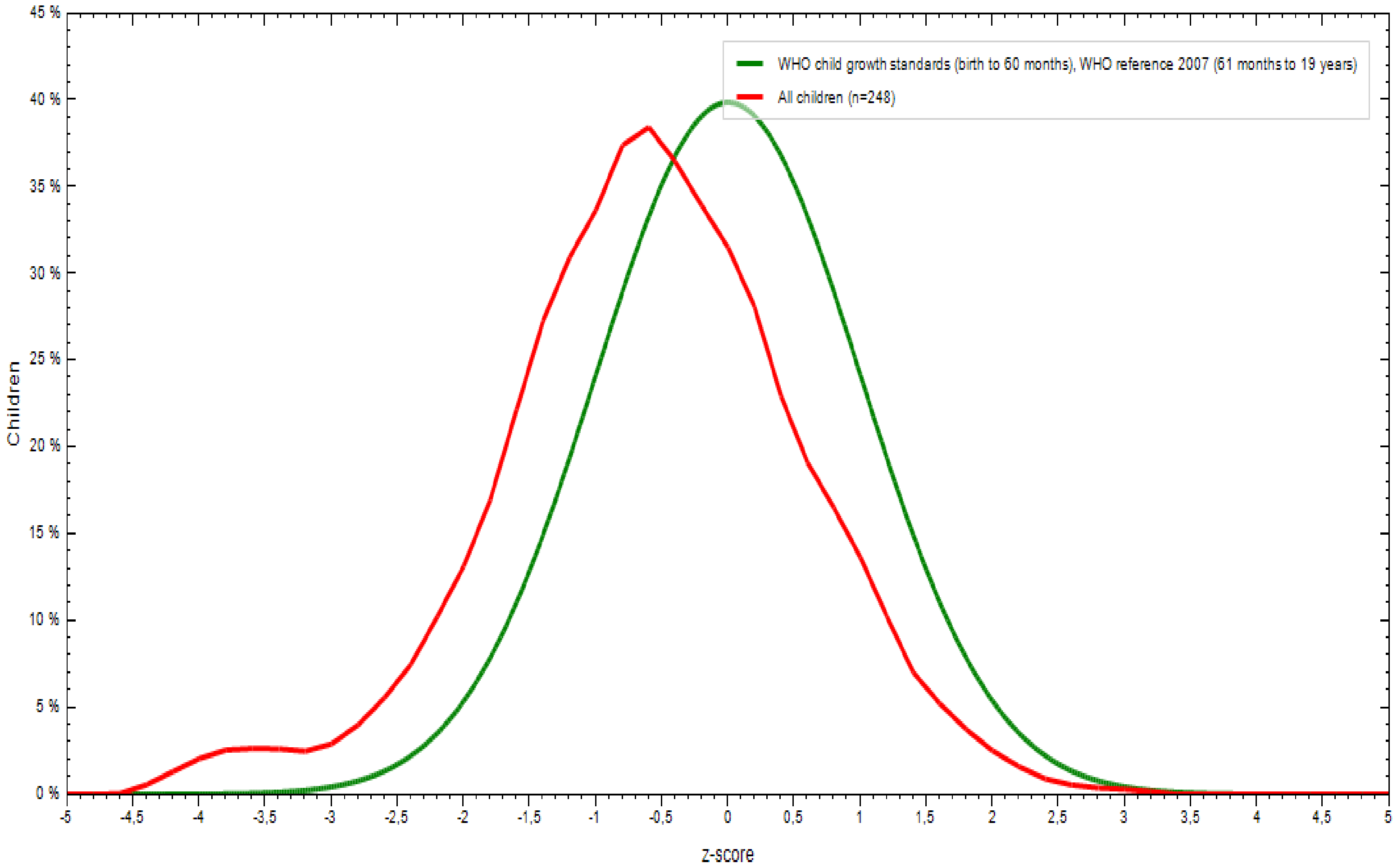

Figure S2. Comparison of participants height-for-age values with WHO growth standards.

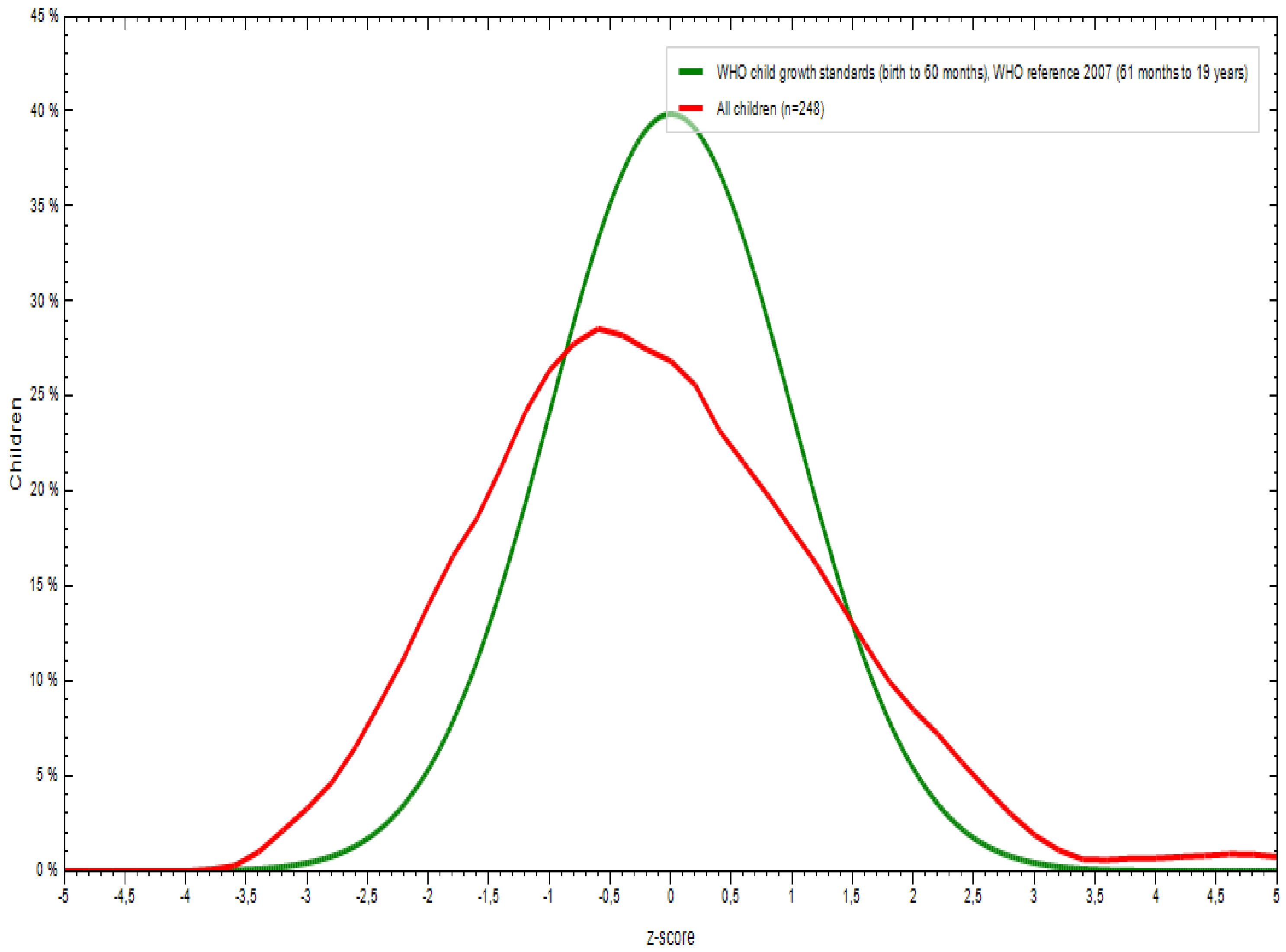

Figure S3. Comparison of participants' weight -for-age values with WHO growth standards.
