# Supplementary material for: A Cross-Sectional Study of the Relationship Between Dietary Micronutrient Intake, Cognition and Academic Performance Among School-Aged Children in Taabo, Côte d’Ivoire
Source: Nutrients. 2025 Nov 18;17(22):3602. doi: 10.3390/nu17223602 (PMC12655121; doi:10.3390/nu17223602)
Supplement: Supplementary file 1 [file nutrients-17-03602-s001.zip › supplementary Table S1..pdf]

Table S1. Mean and standard deviation of nutritional indicators: weight-for-age z-score, height-for-age z-score, and BMI-for-age z-score.

Variable	Mean	Std. Deviation	95% CI Mean Upper	95% CI Mean Lower	Minimum	Maximum
Weight for age	-0.32	1.04	-0.17	-0.46	-2.87	2.77
Height for age	-0.20	1.64	0.01	-0.40	-13.43	5.37
BMI for age	-0.44	2.78	-0.09	-0.78	-3.89	39.61
