# Supplementary material for: A Cross-Sectional Study of the Relationship Between Dietary Micronutrient Intake, Cognition and Academic Performance Among School-Aged Children in Taabo, Côte d’Ivoire
Source: Nutrients. 2025 Nov 18;17(22):3602. doi: 10.3390/nu17223602 (PMC12655121; doi:10.3390/nu17223602)
Supplement: Supplementary file 1 [file nutrients-17-03602-s001.zip › supplementary Table S2.pdf]

Table S2. Mean scores and standard deviation (SD) in cognitive tests, mathematics, and literature and distribution of scores across performance categories: low (below the 50th percentile), medium (50th-75th percentile) and high (above the 75th percentile) performance for cognition test and low below 5/10, medium (5/10-7/10), and high (above 7/10) performance for mathematics and literature.

**Variable**				**Frequency *N* (%)**		
	Mean	±	SD	High	Medium	Low
Cognition	15.4	±	4.4	65 (25.9)	77 (30.7)	109 (43.4)
Mathematics	6.0	±	2.4	67 (26.7)	111 (44.2)	73 (29.1)
Literature	5.4	±	1.8	39 (15.5)	120 (47.8)	92 (36.7)
