# Supplementary material for: A Cross-Sectional Study of the Relationship Between Dietary Micronutrient Intake, Cognition and Academic Performance Among School-Aged Children in Taabo, Côte d’Ivoire
Source: Nutrients. 2025 Nov 18;17(22):3602. doi: 10.3390/nu17223602 (PMC12655121; doi:10.3390/nu17223602)
Supplement: Supplementary file 1 [file nutrients-17-03602-s001.zip › supplementary Table S3.pdf]

Table S3. Comparison of mean scores in cognitive and school performance across nutritional status.

Variable	N	%	Cognition				Mathematics				Literature			
			Mean	±	SD	*p*-value	Mean	±	SD	*p*-value	Mean	±	SD	*p*-value
**Weight for age**														
Underweight	11	8.8	13.1	±	1.7	0.226	4.7	±	3.0[a]	0.035	5.3	±	1.8	0.562
Normal	235	90.8	15.5	±	4.4		6.0	±	2.3[ab]		5.4	±	1.8	
Overweight	5	0.4	15.7	±	5.0		7.8	±	1.4[b]		5.8	±	1.7	
**Height for age**														
Stunting	19	8.8	17.5	±	6.0	0.172	5.6	±	2.6	0.774	11.2	±	3.4	0.188
Normal	214	84.9	15.2	±	4.2		6.0	±	2.3		5.5	±	1.8	
Overgrowth	18	0.4	15.8	±	4.2		5.6	±	2.4		4.9	±	1.5	
**BMI for age**														
Thinness	23	9.2	15.9	±	4.3	0.816	5.9	±	2.5	0.976	5.8	±	2.0	0.570
Normal	224	90	15.3	±	4.4		6.0	±	2.3		5.4	±	1.7	
Overweight/Obesity	2	0.8	17.5	±	7.8		6.3	±	2.4		6.4	±	1.9	

Group comparisons were conducted using the Kruskal–Wallis test. Values sharing the same letter within a column do not differ significantly according to Dunn's post hoc test ($p < 0.05$).
